# P02.71. The effect of acute yoga and aerobic exercise on word memory and anxiety

**DOI:** 10.1186/1472-6882-12-S1-P127

**Published:** 2012-06-12

**Authors:** N Gothe, C Hillman, E McAuley

**Affiliations:** 1 University of Illinois, Urbana Champaign, Urbana, USA

## Purpose

Given the increasing popularity of yoga, exploring its physiological and psychological benefits is important. The purpose of this study was to examine the effects of an acute yoga session, relative to aerobic exercise, on word memory recall and recognition and assess the role of anxiety in improving cognitive performance.

## Methods

A repeated measures design was employed where thirty female college-aged participants (Mage=20.1, sd =1.95) completed three counterbalanced testing sessions: a yoga session, an aerobic exercise session, and a baseline assessment. Participants were presented with the state sub-form of the Spielberger’s State Trait Anxiety Inventory and a list of 40 words immediately after the exercise sessions. They completed a free recall and a recognition task where the 40 words were randomly intermixed with 40 new words.

## Results

Results showed a significant main effect for condition on the number of words correctly recalled, F(2, 27)=14.688, p <.001, partial η2=.521. The number was significantly higher for the yoga condition (M=10.03), as compared to the aerobic (M=6.5, p<.001) and baseline conditions (M=6.87, p<.001). For the word recognition [F(2, 27)=10.032, p<.001, partial η2=.426], participants correctly recognized more words in the yoga condition (M=86.44), as compared to the aerobic (M=74.50, p<.001) and the baseline conditions (M=77.83, p<.02). In addition, the post-exercise state anxiety scores were significantly lower for the yoga session [Myoga=21.77, Maerobic=29.6; t(29)=4.69, p<.001].

## Conclusion

Participation in a 20-minute yoga session resulted in superior memory performance compared to acute aerobic exercise. In line with previous studies, state anxiety was significantly lower after the yoga session. Lower anxiety has shown to be associated with improved performance and it is possible that the mind body elements of yoga reduce anxiety, thereby improving cognitive performance. Overall, these findings highlight the need to assess effects of non-traditional modes of exercise such as yoga on other aspects of cognition and explore their underlying mechanisms.

